# LncRNA LZTS1-AS1 induces proliferation, metastasis and inhibits autophagy of pancreatic cancer cells through the miR-532 /TWIST1 signaling pathway

**DOI:** 10.1186/s12935-023-02979-7

**Published:** 2023-07-04

**Authors:** Hui Wu, Anshu Li, Qichang Zheng, Jingyang Gu, Wei Zhou

**Affiliations:** 1 Research Center, Shanghai Healink Medical Information Consulting Co., LTD, Shanghai, 201102 China; 2 Department of Gastrointestinal Surgery, Union Hospital, Tongji Medical College, Huazhong University of Science and Technology, Wuhan, China; 3 Liver Transplantation Center, Union Hospital, Tongji Medical College, Huazhong University of Science and Technology, Wuhan, China; 4 Department of Pancreatic Surgery, Wuhan No.1 Hospital, No. 215 Zhongshan Road, Qiaokou District, Wuhan, 430022 Hubei China

**Keywords:** Autophagy, lncRNA LZTS1-AS1, miR-532, Pancreatic cancer

## Abstract

The 5 year survival rate after diagnosis of pancreatic cancer (PANC) is less than 5%, and it is one of the malignant tumors with the worst prognosis. Identification of novel oncogenes involved in the occurrence of pancreatic cancer is of great significance to improve the overall survival of PANC patients. Our previous study found that miR-532 is a key factor in PANC occurrence and development, and this study further explored its mechanism. We found that the expression of lncRNA LZTS1-AS1 was elevated in PANC tumor tissues and cells, and correlated with poor prognosis. In vitro experiments confirmed that LZTS1-AS1 could promote proliferation, oncogenicity, migration, and invasion of PANC cells, and inhibit apoptosis and autophagy. However, miR-532 had the completely opposite effect, and inhibition of miR-532 counteracted the effect of LZTS1-AS1 on PANC cells. Dual luciferase gene reporter assay and RNA immunoprecipitation assay confirmed the targeting relationship between LZTS1-AS1 and miR-532, and their expression levels were negatively correlated in PANC tissues. Overexpression of TWIST1 could counteract the effect of miR-532 in PANC cells, and the expression levels of both were negatively changed in PANC tissues and cells. Our results suggest that lncRNA LZTS1-AS1 acts as an oncogene to promote the metastasis of PANC and inhibit autophagy, and its mechanism may be to regulate TWIST1 through sponge miR-532. This study provides novel biomarkers and therapeutic targets for PANC.

**Supplementary Information:**

The online version contains supplementary material available at 10.1186/s12935-023-02979-7.

## Introduction

Pancreatic cancer (PANC) is one of the most common fatal gastrointestinal malignancies. It is still a major global public health problem for its 5 year survival rate is less than 5% [1]. Late diagnosis, aggressive tumor growth, high metastasis and recurrence, and chemotherapy resistance lead to worse therapeutic effects and poor prognosis of PANC [2–4]. It is common in pancreatic cancer to have genetic and epigenetic abnormalities, as well as aberrant activation of tumor-driver genes [5, 6]. Therefore, it is of great significance to identify new oncogenes involved in pancreatic carcinogenesis and improve the early screening and diagnosis rate for improving the overall survival of PANC patients.

The miRNAs are a group of small non-coding RNAs that modify mRNA translation or stability to regulate their target genes [7]. Since their discovery in 1993, miRNAs have been extensively studied for their role in tumor progression. There has been evidence that long non-coding RNAs (lncRNAs) are involved in cancer regulation via sponge miRNAs, interacting with downstream mRNA to create a regulatory network [8–10]. Previous studies have found that miR-532 plays a regulatory role in a variety of nausea tumors, but its direction of action on cancer is divergent. In breast cancer and gastric cancer, miR-532 promotes cancer cell proliferation and metastasis [11, 12], but it inhibits cancer cell proliferation and metastasis in lung cancer and prostate cancer [13–15]. In colorectal cancer (CRC), miR-532 has been reported as a proto-oncogene and tumor suppressor [16, 17].

The role of miR-532 in PANC has not been reported. Alizadeh Savareh B et al. [18] constructed a machine-learning method to determine the diagnostic model of pancreatic cancer using circulating microRNA signatures. According to the statistical analysis of the survival rate, miR-532 was considered to have a meaningful correlation with the prognosis of pancreatic cancer patients. Our previous study found that miR-532 may play a tumor suppressor role in PANC. In this study, we will explore its upstream and downstream mechanism, and explore its potential value in the diagnosis and treatment of PANC.

## Materials and methods

### PANC tissues and cell lines

Seventeen pairs of pancreatic cancer tissues and adjacent normal pancreatic tissues that underwent surgical treatment in Union Hospital, Tongji Medical College, Huazhong University of Science and Technology from January 2020 to February 2022 were collected. All tumor tissues were confirmed as adenocarcinoma by histological identification. All patients understood that the collected tissue would be used for scientific research and provided written informed consent.

Human pancreatic cancer cell lines PANC03.27, Capan-1, Capan-2, SW1990, hPAF-II, Panc 10.05, BXPC-3, CFPAC-1 and immortalized human pancreatic ductal epithelial cell line (HPNE) were purchased from the Cell Bank of Chinese Academy of Sciences (China, Shanghai). Cells were cultured in DMEM supplemented with 10% inactivated FBS (Gibco, USA), 1 × 10^5^ U/L penicillin, and 100 mg/L streptomycin (Gibco). The cells were incubated at 37 °C in a humidified atmosphere of 5% CO_2_. HPNE cells were grown in RPMI-1640 medium (Gibco) supplemented with 10% inactivated fetal bovine serum (FBS).

### Cell transfection

Obio Technology Corp (China) provided the lentivirus constructing TWIST1 overexpress. SiRNA for knockdown LZTS1-AS1 and all the miR-532 mimics, inhibitors, and negative control used for transfection were purchased from GenePharma (Shanghai, China). Transfections were performed according to the manufacturer’s instructions with Lipofectamine 2000 (Invitrogen, USA) (Additional file 1).

### Quantitative RT-PCR

According to the manufacturer's protocol, total RNA was extracted from crushed tissue or cultured cells using a Trizol reagent (Invitrogen). For RT-PCR, 1 g cDNA and SYBR Green RT-PCR kit (Takara, Japan) were used, and an RNA reverse transcription kit (Takara) was used for reverse transcription. For the reaction, 95 °C was pre-denaturated for 3 min, followed by 40 cycles (30 s at 95 °C, 45 s at 58 °C), followed by a 6 min extension at 72 °C. The primer sequences of the primers used were provided in Additional file 2: Table S1. They were synthesized by Shanghai Sangon Biotech Co., Ltd. (China). The 2^−ΔΔCt^ method was used to calculate the relative expression of targets. Repeat the calculation for each sample at least 3 times.

### CCK-8 assay

In accordance with the instructions of the supplier, the CCK-8 kit was purchased from Boster Biological Technology Co., Ltd. (China). Cells in the logarithmic growth phase were seeded into 96-well plates at a concentration of 5 × 10^3^ per well. The optical concentration (OD) values at 450 nm were measured before and after 72 h of culture with a microplate analyzer. Repeat at least 3 times for each sample.

### Cell apoptosis and cell cycle assays

A dye combination of Annexin V-fluorescein isothiocyanate (FITC) and propidium iodide (PI) was used to stain transfected cells (BD Pharmingen, USA) after transfection. A flow cytometry analysis (BD Biosciences, CA, USA) was performed on annexin V + cells. Cells were harvested and fixed with 70% cold ethanol at − 20 °C for cell cycle analysis. Following that, cells were stained with the Cell Cycle Kit (BD Pharmingen) following the manufacturer's instructions.

### Western blot assay

In this study, cells were collected, disrupted with RIPA cleavage buffer (Thermo Fisher Scientific, USA), centrifuged, and the protein concentration was measured with BCA Kit (Thermo Fisher Scientific). SDS-PAGE was performed on 50 mg of protein followed by transfer to PVDF membranes (Millipore, USA). The membranes were blocked in 5% non-fat milk at room temperature for 1 h and then treated overnight at 4 °C according to the antibody protocol described above. The following antibodies purchased from Protientech (USA) were used: anti-ATG5 (66744-1-Ig), anti-ATG12 (67341-1-Ig), anti-LC3 (14600-1-AP), anti-Beclin1 (11306-1-AP), anti-p62 (18420-1-AP); anti-E-cadherin (20874-1-AP), anti-N-cadherin (66219-1-Ig), anti-Vimentin (10366-1-AP), anti-α-SMA (67735-1-Ig) and anti-GAPDH (60004-1-Ig). Moreover, the respective Goat anti-rabbit IgG secondary antibody (Abcam, ab205718) was used to incubate these membranes according to protocol. In order to quantify the protein blots, the Thermo Fisher Scientific ECL system was used in conjunction with Image J software to analyze the data.

### Colony formation assay

Transfected cells were seeded into 6-well plates at 700 cells per well and maintained in DMEM containing 10% FBS for two weeks. A 30 min staining with 0.1% crystal violet followed by imaging and counting was performed after the cells had been fixed with methanol.

### Immunohistochemistry

A 0.3% V/V solution of hydrogen peroxide was used to block the endogenous peroxide activity within cells. Then the cells were incubated with anti-Cyclin D1 (Proteintech, 26939-1-AP) or anti-MMP-9 (Proteintech, 10375-2-AP) overnight at 4 °C, and then incubated with the corresponding biotinylated secondary antibodies. As a counterstain, hematoxylin was used to counterstain the cells after the DAB reactions were developed using the DAB Kit (BD Bioscience, USA). In order to score staining, relative quantitative methods were used based on the intensity and the rate of positive staining.

### Xenografts in nude mice

The CFPAC-1 cells were stably transfected. BALB/C mice (4 weeks old, 18–22 g) were injected subcutaneously with around 1 × 10^7^ cells. Measurements of the tumor size (W) and length (L) were taken every week, and the tumor volume (V) was calculated using the equation V = (W^2^ × L)/2. The entire experimental procedure lasted for 4 weeks, and mice that died during this period were not included in the study. The mice were sacrificed after 4 weeks, and tumors were removed and weighed. The animal studies were performed in accordance with the institutional ethics guidelines for animal experiments, which were approved by the animal management committee of Union Hospital, Tongji Medical College, Huazhong University of Science and Technology (2021 IEC No.228).

### Transmission electron microscopy (TEM)

CFPAC-1 cells were fixed in 2.5% glutaraldehyde without washing at 37 °C and further fixed with 2% osmium tetroxide buffer. The fixed cells were then dehydrated with graded ethanol series and embedded in Epon. Electron microscopy was performed using a Talos F200X S/TEM transmission electron microscope (Thermo Fisher Scientific).

### Transwell assay

600 μl culture medium containing 10% FBS was added to the lower chamber. A single cell suspension containing 8 × 10^4^ cells was seeded on a 200 μl serum-free medium for cell migration and 200 μl matrix gel for cell invasion in the upper chamber. After 24 h of culture, cells in the upper chamber were fixed with 4% paraformaldehyde for 5 min, stained with 0.1% crystal violet for 5 min, and transmembrane cells were counted under a light microscope (Olympus, Japan).

### Dual-luciferase reporter assays

The wild-type (wt) and mutant (mut) 3′-UTR of LZTS1-AS1 were constructed by GenePharma. The luciferase construct and miR-532 mimic were co-transfected into CFPAC-1 cells following the manufacturer's protocol. 48 h after transfection, the luciferase activity was detected by a dual luciferase reporter kit (Promega). Each experiment was repeated 3 times.

### RNA immunoprecipitation (RIP) assay

After being washed using PBS twice, CFPAC-1 cells were lysed with RIPA lysis buffer (ThermoFisher Scientific, USA) containing a protease inhibitor cocktail. The lysate was then incubated with magnetic beads of human anti-Ago2 antibody (Proteintech, 67934-1-Ig) and normal rabbit IgG as control. The beads were washed twice with 700 mM NaCl wash buffer. RNA in immunoprecipitates was isolated using TRIzol reagent (Invitrogen) and subjected to qRT-PCR analysis.

### Statistical analysis

All data are presented as means ± standard deviation (SD). Statistical significance analysis and statistical graphing of the data were performed using Prism GraphPad 8.0 software. One-way ANOVA and Student’s t-test were used for data analysis. Kaplan–Meier curve and log-rank test were used to analyze the survival of patients. Expression correlation analysis was performed using Pearson’s correlation coefficient. P < 0.05 was regarded as a statistically significant difference.

## Results

### LncRNA LZTS1‐AS1 is up-regulated in PANC and is associated with poor prognosis

**Fig. 1:**
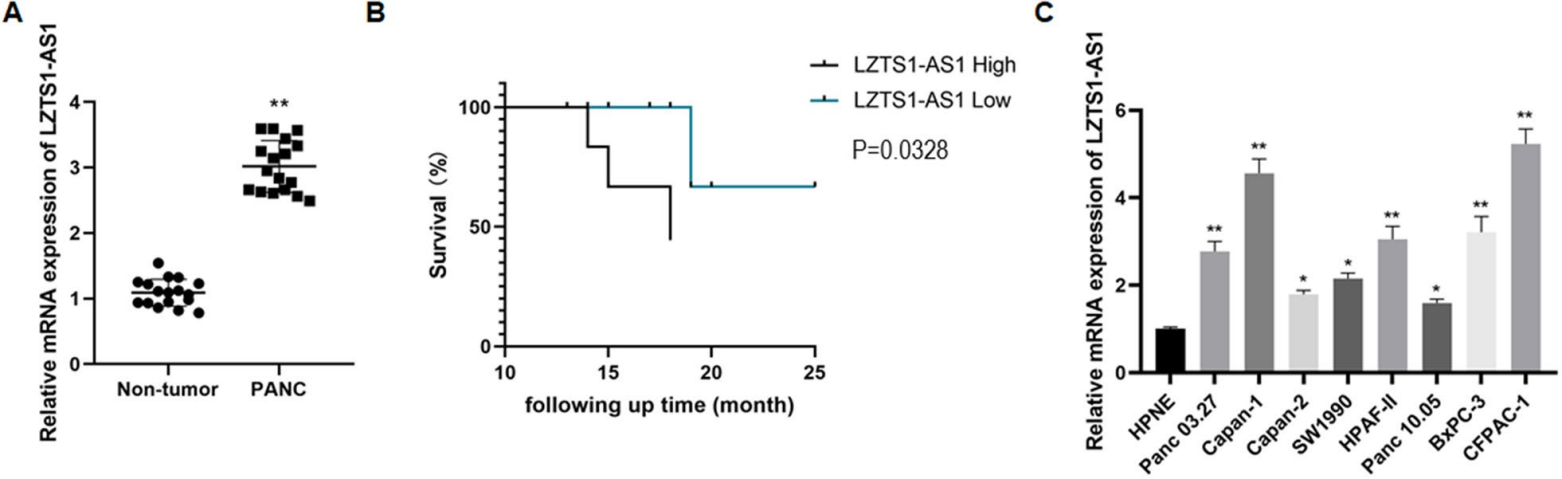
LncRNA LZTS1-AS1 was highly expressed in PANC tissues and cells and predicted a lower survival rate; **A**: The expression level of lncRNA LZTS1-AS1 was significantly increased in PANC tissues, **P < 0.01 compared with Non-tumor; **B**: Survival curves of patients with different lncRNA LZTS1-AS1 expression levels (P = 0.0328); **C**: The expression level of lncRNA LZTS1-AS1 was significantly increased in PANC cell lines, *P < 0.05 and **P < 0.01 compared with HPNE

In this study, tumor and non-tumor tissues of 17 patients with pancreatic cancer who underwent surgical treatment at our hospital from January 2020 to February 2022 were collected. The expression level of lncRNA LZTS1‐AS1 was significantly increased in PANC tumor tissues as detected by qRT-PCR (Fig. 1A, P < 0.01). According to the relative expression of lncRNA LZTS1‐AS1, we divided patients into high and low-expression groups and plotted survival curves separately. It was found that patients with high lncRNA LZTS1‐AS1 expression had a lower survival rate (Fig. 1B, P = 0.0328). Table 1 showed the correlation between the expression level of LZTS1‐AS1 in PANC tumor tissues and the pathological characteristics of patients. Statistical analysis found that the expression level of LZTS1-AS1 was significantly correlated with the TNM stage, tumor size, and lymphatic metastasis of PANC.

**Table 1:** Correlation between lncRNA LZTS1‐AS1 expression and clinicopathological features of pancreatic cancer patients

Parameters	Number of cases	LZTS1‐AS1 expression		P value
		High	Low	
All cases	17	7	10	
Age (years)				
< 65	4	1	3	0.569
≥ 65	13	6	7	
Gender				
Male	12	6	6	0.466
Female	5	1	4	
TNM stage				
I&II	11	2	9	0.023
III&IV	6	5	1	
Histological grade				
Good	4	0	5	0.262
Moderate	8	3	5	
Poor	5	4	0	
Tumor size (cm)				
< 3	8	0	8	< 0.001
≥ 3	9	7	2	
Lymphatic metastasis				
Negative	11	1	10	< 0.001
Positive	6	6	0	

Normal pancreatic cells (HPNE) and pancreatic cancer cells (PANC03.27, Capan-1, Capan-2, SW1990, hPAF-II, Panc 10.05, BXPC-3, CFPAC-1) were also collected in the study. QRT-PCR was used to detect the relative expression level of lncRNA LZTS1-AS1 in each cell line. Compared with the HPNE cells, the expression level of lncRNA LZTS1-AS1 was significantly increased in all pancreatic cancer cell lines (Fig. 1A, P < 0.05 or P < 0.01), of which CFPAC-1 and Capan-1 were the most prominent.

### LZTS1‐AS1 promotes the proliferation, migration, invasion, and oncogenicity of PANC cells, and inhibits apoptosis and autophagy

**Fig. 2:**
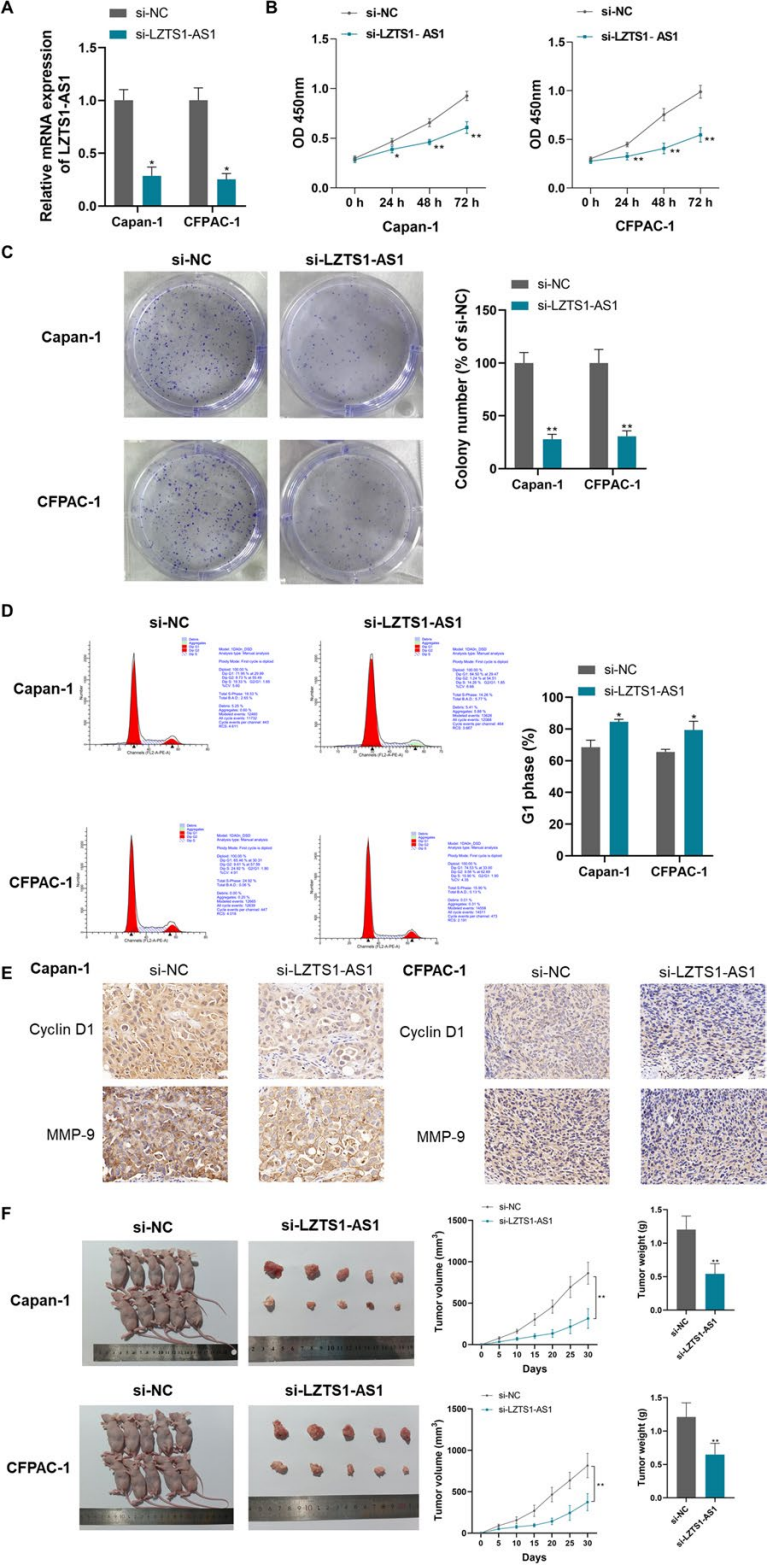
LZTS1‐AS1 promotes the proliferation and oncogenicity of PANC cells; **A**: LZTS1‐AS1 expression was effectively knocked down by siRNA transfection in CFPAC-1 and Capan-1 cells; **B**: Knockdown of LZTS1-AS1 in CFPAC-1 and Capan-1 cells significantly inhibited cell proliferation; **C**: Knockdown of LZTS1-AS1 in CFPAC-1 and Capan-1 cells significantly inhibited cell colony formation; **D**: Knockdown of LZTS1-AS1 in CFPAC-1 and Capan-1 cells resulted in cell cycle arrest at G1 phase; **E**: Knockdown of LZTS1-AS1 significantly inhibited the expression of Cyclin D1 and MMP-9 in CFPAC-1 and Capan-1 cells; **F**: Knockdown of LZTS1-AS1 in CFPAC-1 and Capan-1 cells significantly inhibited the oncogenicity of cells; *P < 0.05 and **P < 0.01 compared with si-NC

To investigate the effect and mechanism of LZTS1-AS1 on PANC cells, si-LZTS1‐AS1 was constructed and transfected into CFPAC-1 and Capan-1 cells in this study. As shown in Fig. 2A, si-LZTS1‐AS1 transfection effectively knocked down the expression level of LZTS1‐AS1 in CFPAC-1 and Capan-1 cells (P < 0.05). Cell proliferation was determined using CCK-8, and it was found that the knockdown of LZTS1‐AS1 significantly inhibited cell proliferation in both cell lines (Fig. 2B, P < 0.01). The results of the colony formation assay also showed that the knockdown of LZTS1‐AS1 significantly inhibited colony formation of both CFPAC-1 and Capan-1 cells (Fig. 2C, P < 0.01). Flow cytometry analysis showed that the knockdown of LZTS1-AS1 caused cell cycle arrest in the G1 phase (Fig. 2D, P < 0.05). Immunohistochemical staining showed that the knockdown of LZTS1-AS1 inhibited the expression of Cyclin D1 and MMP-9 (Fig. 2E). In this study, the oncogenicity of PANC cells with LZTS1-AS1 knockdown was detected by xenografts in nude mice. As shown in Fig. 2F, the tumor volume and weight formed by CFPAC-1 and Capan-1 cells with LZTS1-AS1 knockdown in nude mice were significantly reduced (P < 0.01).

**Fig. 3:**
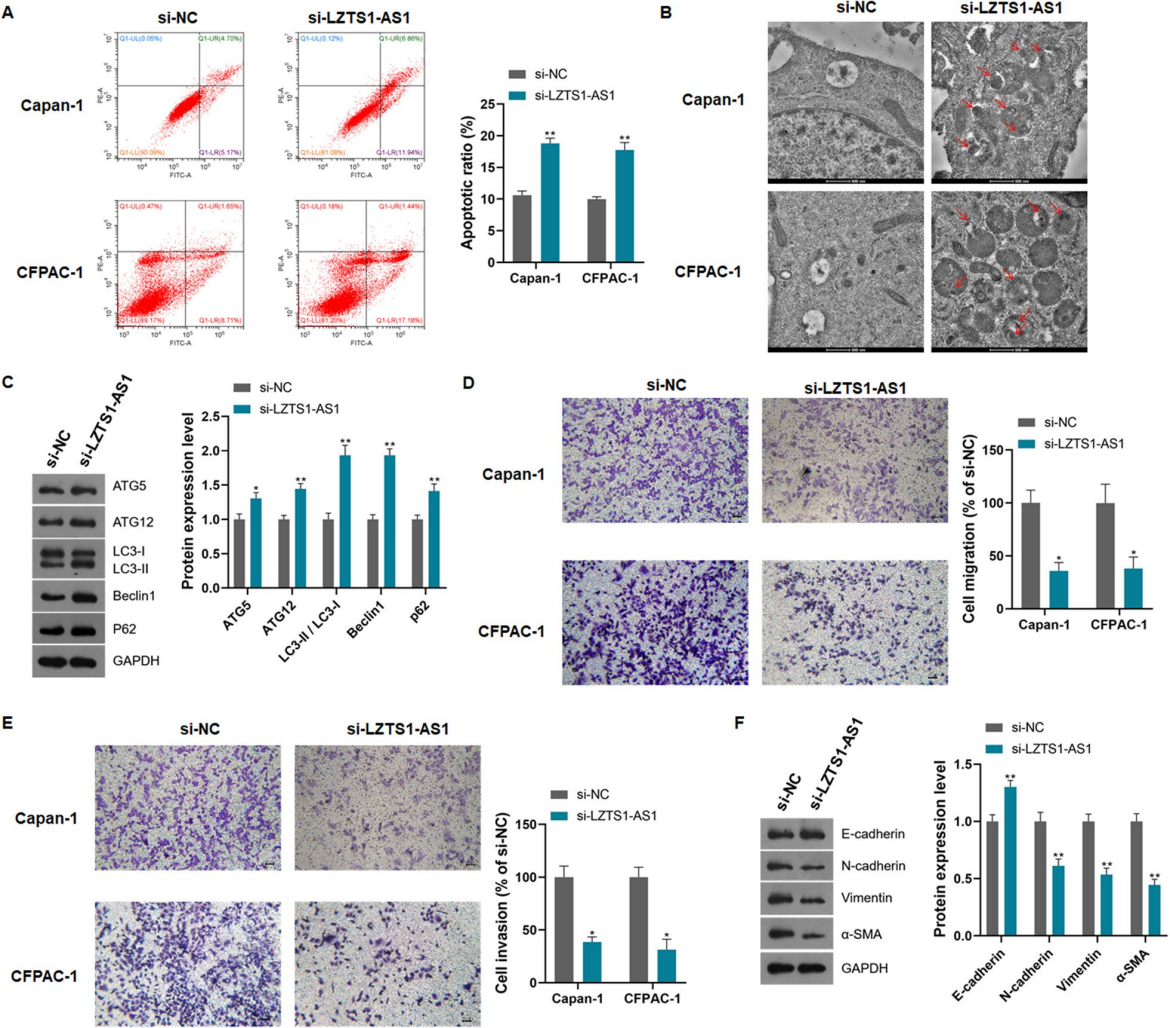
LZTS1‐AS1 promotes migration and invasion of PANC cells, and inhibits apoptosis and autophagy; **A**: Knockdown of LZTS1-AS1 in CFPAC-1 and Capan-1 cells induced apoptosis; **B**: Knockdown of LZTS1-AS1 in CFPAC-1 and Capan-1 cells induced autophagosome formation, red arrows indicate autophagosomes; **C**: Knockdown of LZTS1-AS1 in CFPAC-1 and Capan-1 cells induced the expression of autophagy marker proteins; **D**: Knockdown of LZTS1-AS1 in CFPAC-1 and Capan-1 cells inhibited cell migration; **E**: Knockdown of LZTS1-AS1 in CFPAC-1 and Capan-1 cells inhibited cell invasion; **F**: Knockdown of LZTS1-AS1 in CFPAC-1 and Capan-1 cells inhibited the expression of EMT marker proteins; *P < 0.05 and **P < 0.01 compared with si-NC

Flow cytometry analysis showed that the knockdown of LZTS1-AS1 significantly increased the apoptotic rate of CFPAC-1 and Capan-1 cells (Fig. 3A, P < 0.01). TEM scanning showed that LZTS1-AS1 knockdown induced the formation of autophagosomes in CFPAC-1 and Capan-1 cells (Fig. 3B). Western blot analysis showed that knockdown of LZTS1-AS1 induced the expression of ATG5, ATG12, LC3, Beclin1, and p62 in CFPAC-1 and Capan-1 cells (Fig. 3C, P < 0.05 or P < 0.01). Transwell assay showed that LZTS1-AS1 knockdown significantly inhibited the migration and invasion of CFPAC-1 and Capan-1 cells (Fig. 3D, E, P < 0.05). Western blot was used to detect the expression levels of epithelial-mesenchymal transition (EMT) marker proteins. It was found that knockdown of LZTS1-AS1 significantly increased the expression of E-cadherin and decreased the expression of N-cadherin, Vimentin, and α-SMA in CFPAC-1 and Capan-1 cells (Fig. 3F, P < 0.01).

### LncRNA LZTS1‐AS1 sponges miR-532 in PANC

**Fig. 4:**
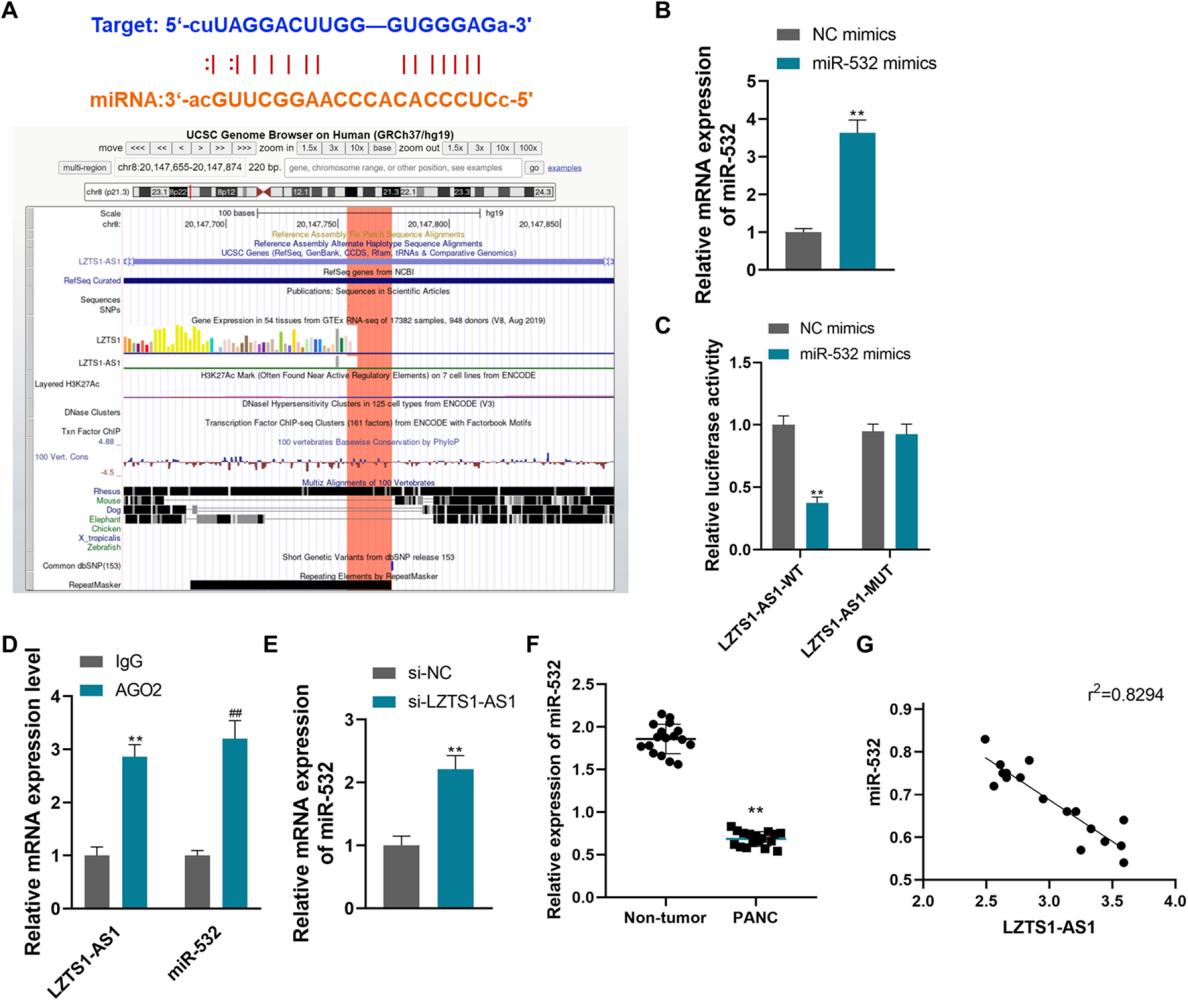
LncRNA LZTS1‐AS1 sponges miR-532 in PANC; **A**: The binding sequence and interaction site of LZTS1-AS1 and miR-532 predicted by starBase; **B**: Transfection with miR-532 mimics effectively overexpressed miR-532 in CFPAC-1 cells, **P < 0.01 compared with NC mimics; **C**: Dual luciferase assay verified the targeting relationship between LZTS1-AS1 and miR-532 in CFPAC-1 cells, **P < 0.01 compared with NC mimics; **D**: RIP assay showed that LZTS1-AS1 and miR-532 were enriched in the compounds precipitated by anti-AgO2 antibody, **P < 0.01 and ##P < 0.01 compared with IgG; **E**: Knockdown of LZTS1-AS1 in CFPAC-1 cells significantly increased the expression level of miR-532, **P < 0.01 compared with si-NC; **F**: The expression of miR-532 was significantly decreased in PANC tissues, **P < 0.01 compared with Non-tumor; **G**: LZTS1-AS1 was negatively correlated with miR-532 expression in PANC tissues, r^2^ = 0.8349

The miRNA that LZTS1-AS1 might target was predicted by the starBase website, and the interaction site between LZTS1-AS1 and miR-532 was found. The predicted binding sequences and interaction sites are shown in Fig. 4A. MiR-532 mimic was constructed and transfected into CFPAC-1 cells, which could effectively increase the expression level of miR-532 (Fig. 4B, P < 0.01). The dual luciferase gene report showed that the luciferase activity of wild-type LZTS1-AS1 was significantly decreased in miR-532 mimic-transfected cells, suggesting that miR-532 had a binding site with LZTS1-AS1 (Fig. 4C, P < 0.01). RIP assay showed that LZTS1-AS1 and miR-532 were enriched in the compounds precipitated by anti-AgO2 antibody (Fig. 4D, P < 0.01). Knockdown of LZTS1-AS1 by siRNA transfection in CFPAC-1 cells significantly increased the expression level of miR-532 (Fig. 4E, P < 0.01). The above results confirmed the sponge effect of LZTS1-AS1 on miR-532 in PANC cells.

Detection of miR-532 expression in PANC tissues and non-tumor tissues showed that the expression of miR-532 was significantly decreased in PANC tissues (Fig. 4F, P < 0.01). Correlation analysis showed that the expression level of miR-532 was negatively correlated with LZTS1-AS1 in PANC tumor tissues (Fig. 4G, r^2^ = 0.8349, 95% confidence interval (CI): − 0.9677 ~ − 0.7649, P < 0.001).

### LZTS1‐AS1 promotes PANC cell proliferation, oncogenicity, migration, and invasion, and inhibits cell apoptosis and autophagy through sponge miR-532

**Fig. 5:**
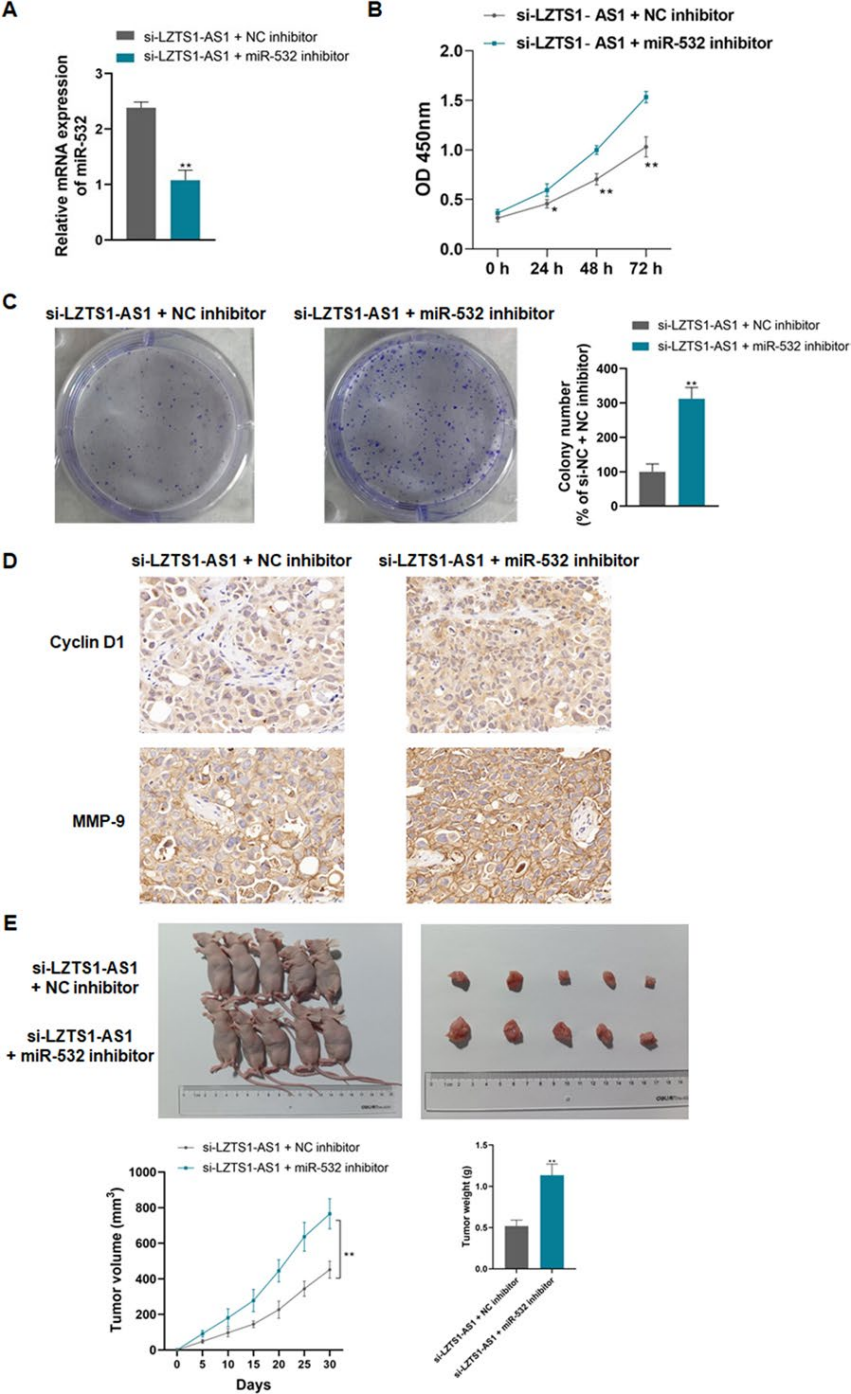
LZTS1‐AS1 promotes the proliferation and oncogenicity of PANC cells through sponge miR-532; **A**: The miR-532 inhibitor effectively knocked down the expression level of miR-532 in CFPAC-1 cells; **B**: Compared with LZTS1-AS1 knockdown alone, simultaneous inhibition of miR-532 significantly increased cell proliferation; **C**: Compared with LZTS1-AS1 knockdown alone, simultaneous inhibition of miR-532 significantly increased cell colony formation; **D**: Compared with LZTS1-AS1 knockdown alone, simultaneous inhibition of miR-532 significantly increased the expression of Cyclin D1 and MMP-9; **E**: Compared with LZTS1-AS1 knockdown alone, simultaneous inhibition of miR-532 significantly increased tumor volume and weight in nude mice; **P < 0.01, compared with si-LZTS1-AS1 + NC inhibitor

CFPAC-1 cells were simultaneously transfected with si-LZTS1-AS1 and miR-532 inhibitors to explore the relationship between LZTS1-AS1 and miR-532 on PANC. The miR-532 inhibitor effectively knocked down the expression level of miR-532 in CFPAC-1 cells (Fig. 5A, P < 0.01). Compared with LZTS1-AS1 knockdown alone, simultaneous inhibition of miR-532 significantly increased cell proliferation, colony formation, and the expression of Cyclin D1 and MMP-9 of CFPAC-1 cells (Fig. 5B–D, P < 0.01). Inhibition of miR-532 significantly increased tumor volume and weight based on LZTS1-AS1 knockdown in nude mice (Fig. 5E, P < 0.01).

**Fig. 6:**
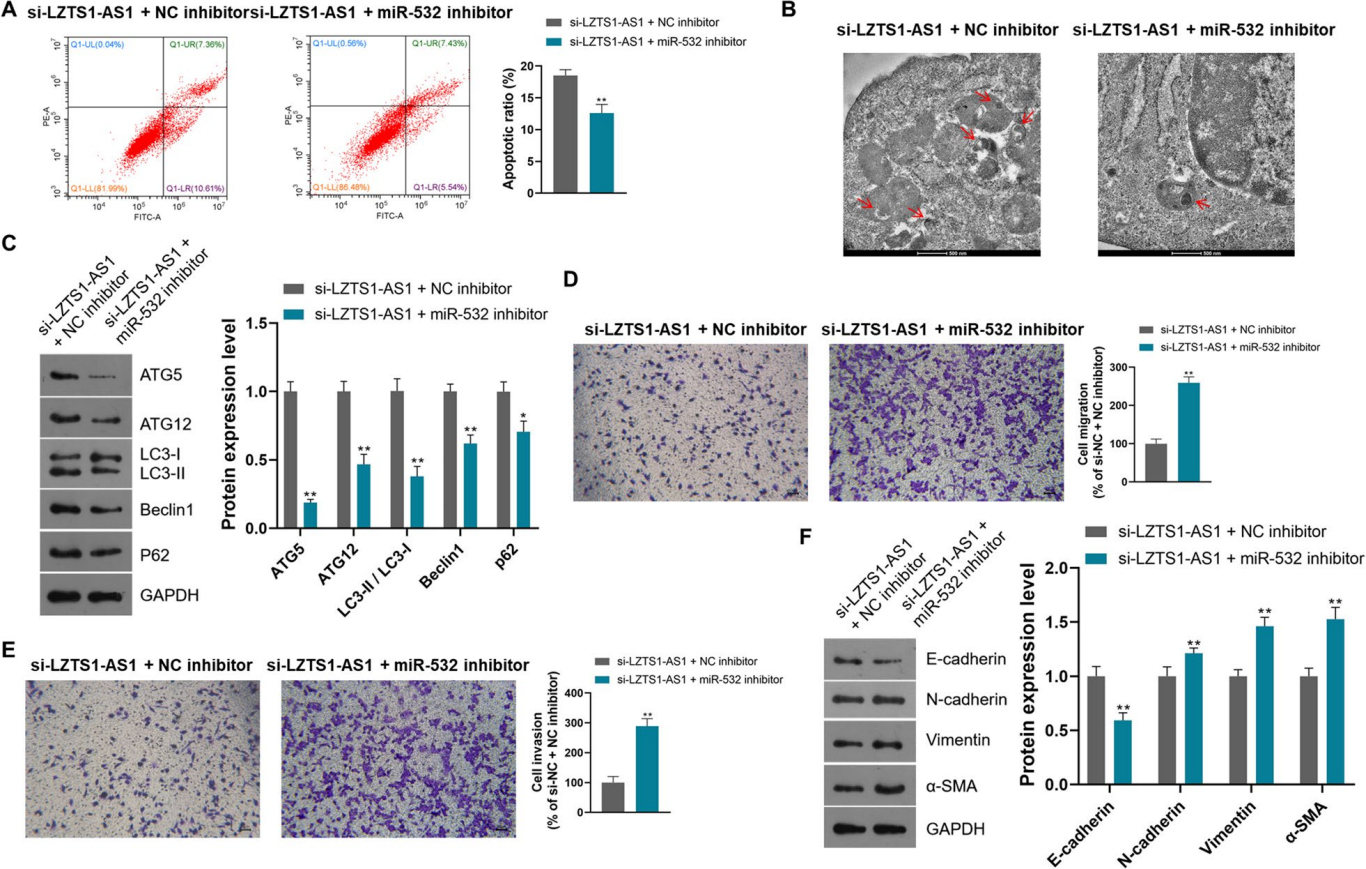
LZTS1‐AS1 promotes PANC cell migration and invasion, and inhibits cell apoptosis and autophagy through sponge miR-532; **A**: Compared with LZTS1-AS1 knockdown alone, simultaneous inhibition of miR-532 significantly suppressed cell apoptosis; **B**: Compared with LZTS1-AS1 knockdown alone, simultaneous inhibition of miR-532 significantly suppressed cell autophagy; **C**: Compared with LZTS1-AS1 knockdown alone, simultaneous inhibition of miR-532 significantly suppressed the expression of autophagy marker proteins; **D**: Compared with LZTS1-AS1 knockdown alone, simultaneous inhibition of miR-532 significantly increased cell migration; **E**: Compared with LZTS1-AS1 knockdown alone, simultaneous inhibition of miR-532 significantly increased cell invasion; **F**: Compared with LZTS1-AS1 knockdown alone, simultaneous inhibition of miR-532 significantly promoted the expression of EMT marker proteins; * P < 0.05 and **P < 0.01, compared with si-LZTS1-AS1 + NC inhibitor

Further studies showed that miR-532 inhibition also significantly inhibited apoptosis, autophagy and autophagy marker proteins expression in CFPAC-1 cells on the basis of LZTS1-AS1 knockdown (Fig. 6A–C, P < 0.01). Similarly, knockdown of LZTS1-AS1 combined with inhibition of miR-532 resulted in higher migration and invasion abilities and EMT marker proteins expression levels than LZTS1-AS1 alone in CFPAC-1 cells (Fig. 6D–F, P < 0.01). The above findings suggest that the effect of LZTS1-AS1 on PANC cells is achieved through sponge miR-532.

### MiR-532 negatively regulates TWIST1 expression in PANC

**Fig. 7:**
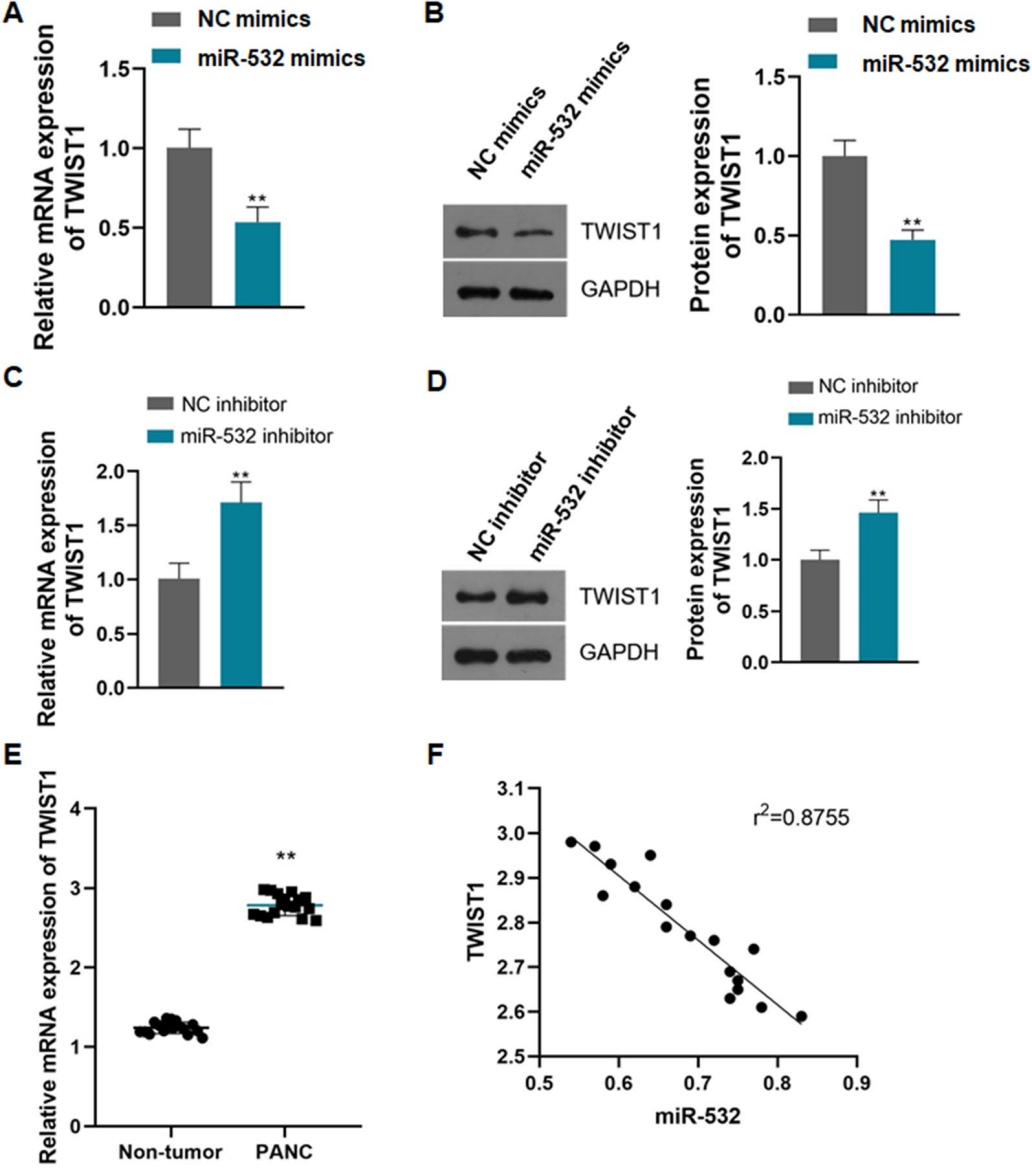
MiR-532 negatively regulates TWIST1 expression in PANC; **A**: Overexpression of miR-532 in CFPAC-1 cells significantly decreased TWIST1 mRNA expression level, **P < 0.01 compared with NC mimics; **B**: Overexpression of miR-532 in CFPAC-1 cells significantly decreased TWIST1 protein expression level, **P < 0.01 compared with NC mimics; **C**: Knockdown of miR-532 in CFPAC-1 cells significantly increased TWIST1 mRNA expression level, **P < 0.01 compared with NC inhibitor; **D**: Knockdown of miR-532 in CFPAC-1 cells significantly increased TWIST1 protein expression level, **P < 0.01 compared with NC inhibitor; **E**: TWIST1 mRNA expression was significantly increased in PANC tissues, **P < 0.01 compared with Non-tumor; **F**: There was a negative correlation between miR-532 and TWIST1 mRNA expression in PANC tissues, r^2^ = 0.8755

CFPAC-1 cells were transfected with miR-532 mimics to overexpress miR-532, and the TWIST1 expression level was detected. It was found that overexpression of miR-532 significantly reduced the mRNA and protein expression levels of TWIST1 (Fig. 7A, B, P < 0.01). Consistent with this, the knockdown of miR-532 using a miRNA inhibitor significantly increased TWIST1 mRNA and protein expression in CFPAC-1 cells (Fig. 7C, D, P < 0.01).

It was found that the expression of TWIST1 mRNA was significantly increased in PANC tissues compared with non-tumor tissues (Fig. 7E, P < 0.01). Correlation analysis showed that the expression level of miR-532 was significantly negatively correlated with TWIST1 in PANC tissues (Fig. 7F, r^2^ = 0.8755, 95% CI = − 0.9770 ~ − 0.8270, P < 0.001).

### MiR-532 inhibits PANC cell proliferation, tumorigenesis, migration, and invasion, and induces apoptosis and autophagy by negatively regulating TWIST1

**Fig. 8:**
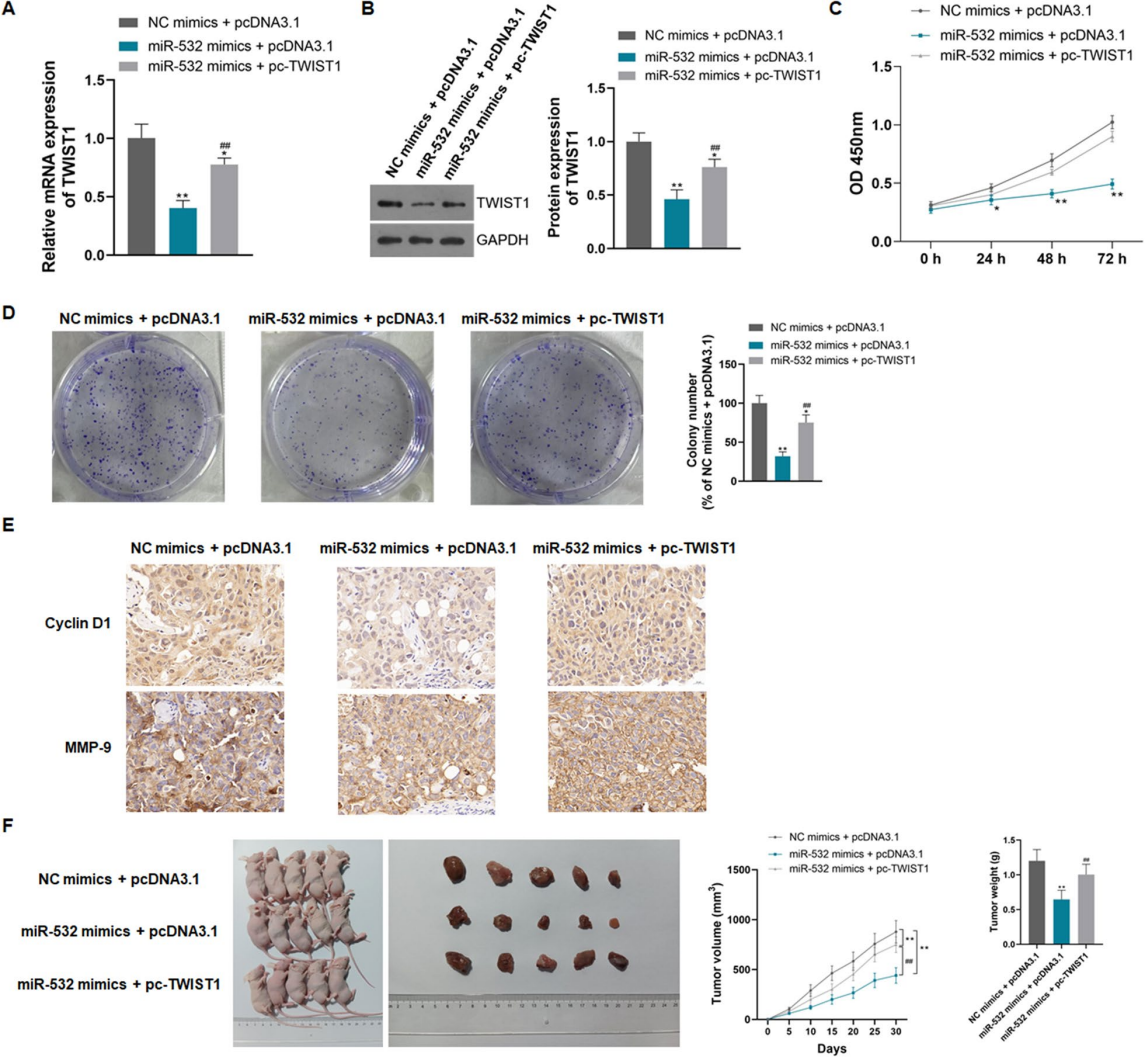
MiR-532 inhibits the proliferation and tumorigenesis of PANC cells by negatively regulating TWIST1; **A**: Transfection of miR-532 mimics significantly reduced the expression level of TWIST mRNA, and TWIST1 plasmid transfection effectively overexpressed TWIST1 mRNA; **B**: Transfection of miR-532 mimics significantly reduced the expression level of TWIST protein, and TWIST1 plasmid transfection effectively overexpressed TWIST1 protein; **C**: Overexpression of TWIST1 reversed the miR-532-inhibited cell proliferation; **D**: Overexpression of TWIST1 reversed the miR-532-inhibited cell colony formation; **E**: Overexpression of TWIST1 reversed the miR-532-inhibited expression of Cyclin D1 and MMP-9; **F**: Overexpression of TWIST1 reversed the miR-532-inhibited cell oncogenicity; *P < 0.05 and **P < 0.01 compared with NC mimics + pcDNA3.1, ##P < 0.01 compared with miR-532 mimics + pcDNA3.1

In this study, based on overexpression of miR-532, TWIST1 overexpression plasmid was constructed and transfected into CFPAC-1 cells to explore the effect and mechanism of miR-532 on PANC. Figure 8A, B showed the relative expression levels of TWIST1 mRNA and protein in each group of cells. MiR-532 mimic transfection could significantly inhibit TWIST expression, and the TWIST1 plasmid could effectively overexpress TWIST1 in CFPAC-1 cells (P < 0.01). Overexpression of miR-532 significantly inhibited cell proliferation, colony formation, and expression of Cyclin D1 and MMP-9, while overexpression of TWIST reversed the effect of miR-532 (Fig. 8C–E, P < 0.01). Cells overexpressing miR-532 had the poor tumorigenic ability and formed smaller tumor volumes and weights in nude mice, while overexpression of TWIST could offset the tumor suppressive effect of miR-532 (Fig. 8F, P < 0.01).

**Fig. 9:**
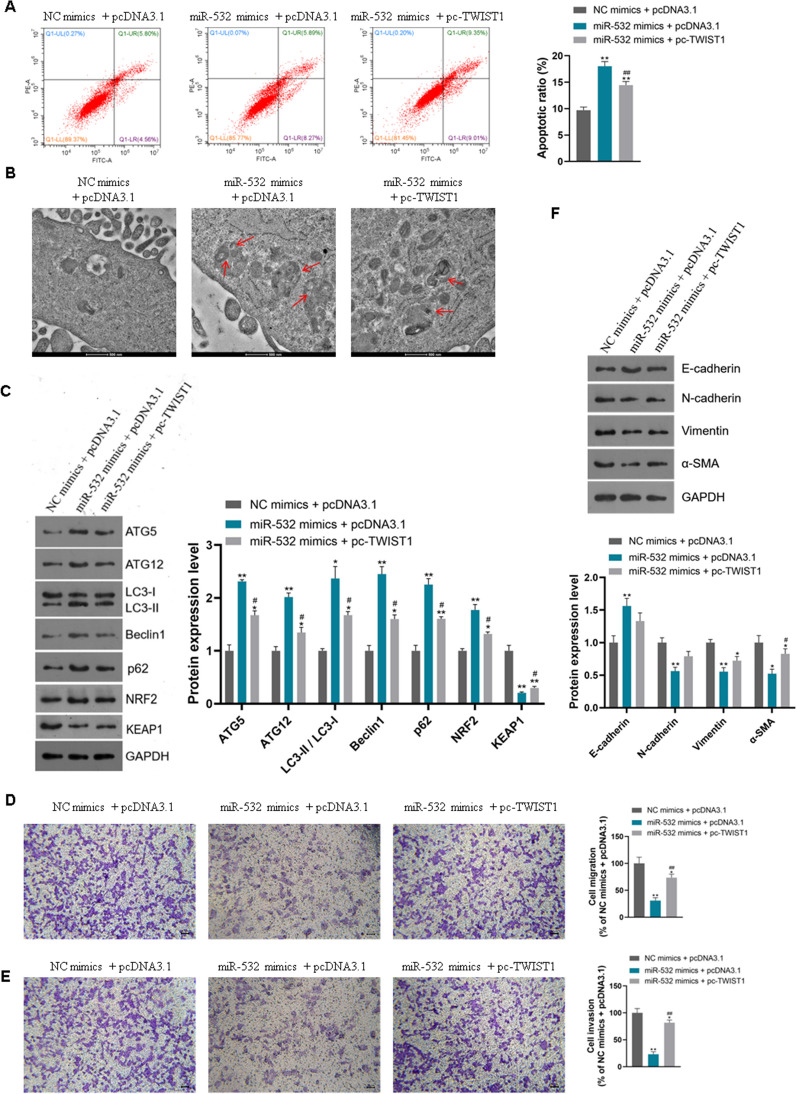
MiR-532 inhibits PANC cell migration and invasion and induces apoptosis and autophagy by negatively regulating TWIST1; **A**: Overexpression of TWIST1 reversed the induction of apoptosis by miR-532; **B**: Overexpression of TWIST1 reversed the induction of autophagy by miR-532; **C**: Overexpression of TWIST1 reversed the induction of expression of autophagy marker proteins by miR-532; **D**: Overexpression of TWIST1 reversed the inhibition of miR-532 on the migration of cells; **E**: Overexpression of TWIST1 reversed the inhibition of miR-532 on the invasion of cell; **F**: Overexpression of TWIST1 reversed the inhibition of miR-532 on the expression of EMT marker proteins; *P < 0.05 and **P < 0.01 compared with NC mimics + pcDNA3.1, #P < 0.05 and ##P < 0.01 compared with miR-532 mimics + pcDNA3.1

**Fig. 10:**
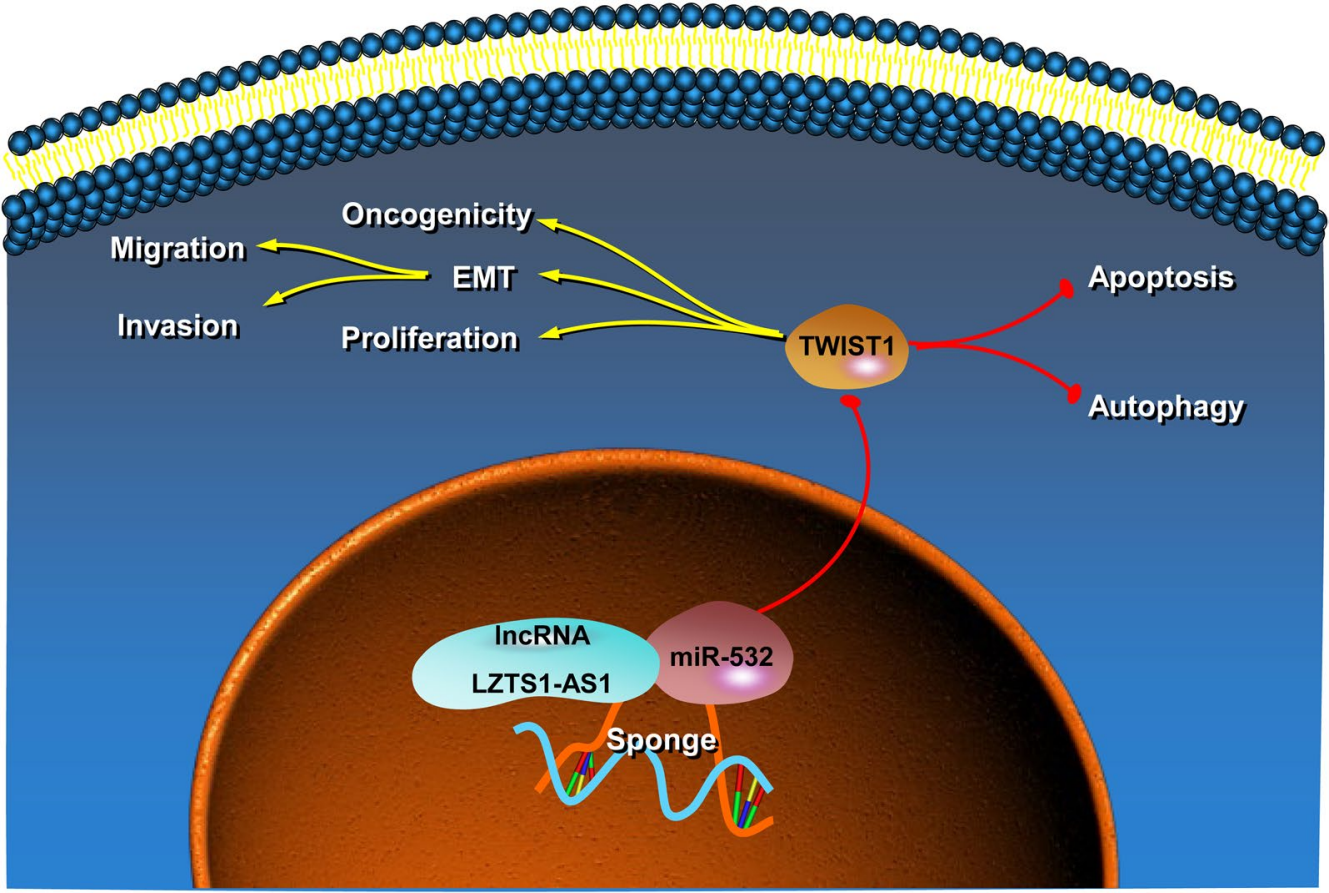
Illustration of the mechanism by which LZTS1-AS1 and miR-532 function in PANC

Besides, miR-532 induced apoptosis and autophagy as well as the expression of autophagy marker proteins in CFPAC-1 cells, and overexpression of TWIST1 could counteract the induction of miR-532 (Fig. 9A–C, P < 0.01). In addition to traditional autophagy proteins, we also detected the expression levels of NFR2 and KEAP1 in CFPAC-1 cells. MiR-532 induced the expression of NFR2 and inhibited the expression of KEAP1, and overexpression of TWIST1 reversed the effect of miR-532 (Fig. 9C, P < 0.05). MiR-532 inhibited the migration and invasion of CFPAC-1 cells and the expression of EMT marker proteins, and overexpression of TWIST1 reversed the inhibitory effect of miR-532 (Fig. 9D–F, P < 0.01). These results suggest that miR-532 inhibits PANC progression by negatively regulating TWIST1 (Fig. 10).

## Discussion

Leucine zipper tumor suppressor 1 (LZTS1) antisense RNA 1 (LZTS1-AS1) is a lncRNA located at Chr8p21.3, which has been reported to be associated with rheumatoid arthritis [19]. Regarding the role of LZTS1-AS1 in pancreatic cancer, the study is the first report. Our study found that LZTS1-AS1 was highly expressed in pancreatic cancer tissues and associated with poor prognosis, and knockdown of LZTS1-AS1 in pancreatic cancer cells significantly inhibited cell proliferation, tumorigenicity, metastasis, and induced autophagy, suggesting its inhibitory effect on pancreatic cancer. These results add LZTS1-AS1 to the range of biomarkers for pancreatic cancer screening.

It has been reported that lncRNA plays a regulatory role in pancreatic cancer [20–22]. Mechanistic studies have found that lncRNA can contribute to the progression of pancreatic cancer by modulating the expression of miRNA to form a lncRNA-miRNA-mRNA regulatory network [23]. In this study, the starBase database was used to predict the binding site of LZTS1-AS1 with miR-532, and the dual luciferase gene reporter assay and RIP assay were used to verify the interaction between LZTS1-AS1 and miR-532 in pancreatic cancer cells. There was a negative correlation between LZTS1-AS1 and miR-532 expression levels in pancreatic cancer tissues. The effect of LZTS1-AS1 knockdown on the biological function of pancreatic cancer cells can be offset by inhibiting miR-532. These results indicate that LZTS1-AS1 promotes pancreatic cancer progression through sponge miR-532.

The regulatory effect of miR-532 on the proliferation and metastasis of cancer cells has been reported in many previous studies [13–15], but its effect on autophagy is rarely explored. It has been reported that miRNA-532 can reverse the inhibitory effect of Rab3IP on the autophagy signaling pathway in gastric cancer [24]. It has also been reported that autophagy-related circular RNA (ACR) is downregulated in chronic heart failure and may suppress hypoxia-induced cardiomyocytes by downregulating miR-532 [25]. In this study, miR-532 was found to inhibit cell proliferation and induce autophagy in pancreatic cancer. Coincidentally, the role of miR-532 in pancreatic cancer is opposite to what has been reported for its role in gastric cancer [24], both in cell metastasis and autophagy.

As early as 2013, it was found that Twist family BHLH transcription factor 1 (TWIST1) was hypermethylated in pancreatic cancer [26]. Recent studies have found that TWIST1 promotes the Warburg metabolism of pancreatic cancer by transcriptionally regulating glycolytic genes [27]. It can be said that TWIST1 plays multiple important roles in the occurrence and development of pancreatic cancer. Not surprisingly, TWIST1 plays a regulatory role in the proliferation, migration, invasion, and EMT of pancreatic cancer cells [28–30], and TWIST1 is involved in the regulation as a miRNA target in these reports. In epithelial ovarian cancer, miR-532 has been identified as a prognostic marker and inhibits cell proliferation and invasion by targeting TWIST1 [31]. Our study found that miR-532 also inhibited cell proliferation and metastasis by targeting TWIST1 in pancreatic cancer.

P62 is a substrate protein of autophagy and a key agonist of NRF2 [32]. Degradation of p62 was reduced in autophagy-deficient cells. When p62 competitively binds KEAP1, NRF2 is released and enters the nucleus to activate downstream target gene transcription and promote tumor cell survival [33]. Our study confirmed that miR-532 induces autophagy in pancreatic cancer cells through TWIST1, and the KEAP1/NRF2 pathway may be its downstream target gene. It has been reported that autophagy deficiency stabilizes TWIST1 protein through SQSTM1/p62 accumulation [34]. SQSTM1 binds to TWIST1 to inhibit the degradation of TWIST1 in autophagosomes and proteasomes. SQSTM1-mediated stable expression of TWIST1 promotes EMT in vitro and promotes tumor growth and metastasis in mice. This may explain the transitional role of TWIST1 between autophagy and EMT. Moreover, TWIST1 has been reported to play an intermediate role between EMT and autophagy in cancers and diabetic kidney disease [35–37]. In this study, TWIST1 was also involved in the regulation of EMT and autophagy in pancreatic cancer. However, whether the relationship among autophagy, EMT, and stable TWIST1 is the key to tumor proliferation and metastasis in pancreatic cancer remains to be further explored.

In conclusion, our study found that lncRNA LZTS1-AS1 is highly expressed in pancreatic cancer tissues and is associated with poor prognosis. It can promote proliferation, metastasis, and oncogenicity and inhibit autophagy of pancreatic cancer cells. Further investigation revealed that LZTS1-AS1 may exert a positive effect on pancreatic cancer by regulating TWIST1 through sponge miR-532. These findings provide a basis for LZTS1-AS1 as a novel biomarker for pancreatic cancer.

## Supplementary Information

**Additional file 1.** The standard curve of CCK-8 assay.

**Additional file 2: Table S1.** Primer sequences used in the study.

## Data Availability

The datasets generated and analyzed during the current study are available from the corresponding author upon reasonable request.

## Abbreviations

PANC: Pancreatic cancer
lncRNAs: Long non-coding RNAs
CRC: Colorectal cancer
FBS: Fetal bovine serum
SDS-PAGE: Sodium dodecyl sulfate–polyacrylamide gel electrophoresis
PVDF: Polyvinylidene difluoride
SD: Standard deviation
EMT: Epithelial-mesenchymal transition
LZTS: Leucine zipper tumor suppressor 1
TWIST: Twist family BHLH transcription factor 1
