# Supplementary material for: LncRNA LZTS1-AS1 induces proliferation, metastasis and inhibits autophagy of pancreatic cancer cells through the miR-532 /TWIST1 signaling pathway
Source: Cancer Cell Int. 2023 Jul 4;23:130. doi: 10.1186/s12935-023-02979-7 (PMC10320869; doi:10.1186/s12935-023-02979-7)
Supplement: Supplementary file 2 — Additional file 2: Table S1. Primer sequences used in the study. [file 12935_2023_2979_MOESM2_ESM.docx]

**Table S1** Primer sequences used in the study

| **Gene name** | **Primer sequences** |
| --- | --- |
| LZST1-AS1 |  |
| miR-532 | Forward: 5’- TGATGAGCATCTGAAGACGGA-3’;  Reverse: 5’- GGAGGCACAAGGAAAGACCAA-3’ |
| TWIST1 | Forward: 5’- GGAGTCCGCAGTCTTACGAG -3’;  Reverse: 5’- TCTGGAGGACCTGGTAGAGG -3’ |
| U6 | Forward: 5’- CTCGCTTCGGCAGCACA -3’;  Reverse: 5’- AACGCTTCACGAATTTGCGT -3’ |
| GAPDH | Forward: 5’-AGCCACATCGCTCAGACAC-3’;  Reverse: 5’-GCCCAATACGACCAAATCC-3’ |
